# A Model to Study the Impact of Polymorphism Driven Liver-Stage Immune Evasion by Malaria Parasites, to Help Design Effective Cross-Reactive Vaccines

**DOI:** 10.3389/fmicb.2016.00303

**Published:** 2016-03-11

**Authors:** Kirsty L. Wilson, Sue D. Xiang, Magdalena Plebanski

**Affiliations:** Vaccines and Infectious Diseases Laboratory, Department of Immunology and Pathology, Central Clinical School, Faculty of Medicine, Nursing and Health Sciences, Monash UniversityMelbourne, VIC, Australia

**Keywords:** circumsporozoite protein, CD8 epitope, altered peptide ligands, polymorphism, cross reactivity, vaccines

## Abstract

Malaria parasites engage a multitude of strategies to evade the immune system of the host, including the generation of polymorphic T cell epitope sequences, termed altered peptide ligands (APLs). Herein we use an animal model to study how single amino acid changes in the sequence of the circumsporozoite protein (CSP), a major target antigen of pre-erythrocytic malaria vaccines, can lead to a reduction of cross reactivity by T cells. For the first time in any APL model, we further compare different inflammatory adjuvants (Montanide, Poly I:C), non-inflammatory adjuvants (nanoparticles), and peptide pulsed dendritic cells (DCs) for their potential capacity to induce broadly cross reactive immune responses. Results show that the capacity to induce a cross reactive response is primarily controlled by the T cell epitope sequence and cannot be modified by the use of different adjuvants. Moreover, we identify how specific amino acid changes lead to a one-way cross reactivity: where variant-x induced responses are re-elicited by variant-x and not variant-y, but variant-y induced responses can be re-elicited by variant-y and variant-x. We discuss the consequences of the existence of this one-way cross reactivity phenomenon for parasite immune evasion in the field, as well as the use of variant epitopes as a potential tool for optimized vaccine design.

## Introduction

Immune evasion by parasites is a major obstacle to overcome for vaccine development. One of the mechanisms employed by malaria parasites is the development of variant epitope sequences, termed altered peptide ligands (APLs). APLs contain multiple or single amino acid changes that can affect their immunogenicity or ability to be recognized by pre-existing immune responses (Plebanski et al., 1997). There are many different APLs in regional populations of parasites, and one possible way to increase cross reactive immunity to APL epitopes is through vaccine design against multiple variant antigens.

Vaccines to malaria, including the recently approved RTS,S (Mosquirix) vaccine, tend to focus on the pre-erythrocytic stage of malaria to prevent infection progressing to the clinically symptomatic erythrocytic stage (Duffy et al., 2012). Liver stage malaria infection by sporozoites would benefit from a CD8 T cell response to eliminate parasitized hepatocytes (Doolan and Martinez-Alier, 2006). Eliciting such a CD8 T cell response is a challenge for vaccine design in particular due to the inability of most adjuvants to induce high levels of CD8 T cells. Furthermore, in addition to high levels of CD8 T cell responses, prospective malaria vaccines would need to induce broad cross reactivity to multiple variant epitopes to ensure protective immunity.

Vaccine adjuvants have the capacity to enhance recognition, and potentially cross reactivity, to vaccine antigens. Nonetheless, this question has not been widely addressed experimentally as yet in any vaccine model. Traditionally adjuvants enhance immune responses by eliciting inflammatory cytokine production. Whilst the most common adjuvant, Alum, induces Th2 biased responses (McKee et al., 2009), certain inflammatory type experimental adjuvants, such as the water-in-oil emulsion, Montanide, and the toll like receptor 3 (TLR3) agonist, Poly I:C, are well regarded for their ability to elicit CD8 T cells to short peptide epitopes (Aucouturier et al., 2002; Elliott et al., 2008; Herrera et al., 2011; Cho et al., 2013). However, not all adjuvant systems need to induce inflammatory cytokines to elicit potent responses. *Ex vivo* peptide pulsed DCs are naturally immunogenic, and do not require the addition of an adjuvant, as they can be directly loaded *ex vivo* with peptides and transferred back to a host, to induce substantial immune responses. Furthermore, novel polystyrene nanoparticles (PSNPs), in the viral size range of 40–50 nm, with covalently bound peptide are capable of inducing robust CD8 T cell responses, in the absence of conventional inflammatory signals (Xiang et al., 2013). In our previous studies the non-inflammatory PSNP vaccines induced comparable levels of CD8 T cell immune responses to peptide delivered with the inflammatory adjuvant Montanide (Wilson et al., 2015). Alongside adjuvant selection, target antigen choice for vaccines is an important consideration for the induction of optimal antigen-specific immunity.

One such target antigen for *Plasmodium falciparum* liver stage malaria vaccine development, the circumsporozoite protein (CSP), displays a wide range of APL variant epitopes. CSP from *P. falciparum* contains two main variable T cell regions, T helper region 2 and 3 (Th2R and Th3R), both of which contain highly polymorphic nested CD4 and CD8 T cell epitopes (Plebanski et al., 1997). In the case of the human leukocyte antigen B35 (HLA-B35) binding region within Th3R, only two of the multiple polymorphic variants bind major histocompatibility complex (MHC) molecules, and naturally induced CD8 T cell reactivity to these two variants is not cross reactive (Gilbert et al., 1998; Plebanski et al., 1999). The current leading pre-erythrocytic vaccine, RTS,S induces CD4 but not CD8 T cells in humans (Lalvani et al., 1999; Reece et al., 2004). It is relevant to note that this vaccine showed better efficacy against the parasites in the population bearing the allelic variant of CSP also present in the vaccine (Neafsey et al., 2015), highlighting the importance of considering antigen polymorphism during vaccine design. Polymorphic antigens pose numerous problems, however, for complex pathogens, such as malaria, polymorphic antigen candidates may not be easily avoided. Therefore, devising strategies to overcome immune evasion and complications with polymorphic antigens for vaccine design is an important question to address in this field.

Mouse models of variant epitopes are a useful tool to understand and demonstrate the impact of amino acid changes to T cell epitopes, even though these epitopes cannot be directly used in human vaccines. The known protective immunodominant CD8 T cell epitope of the murine strain *P. berghei* CSP is SYIPSAEKI (also named pb9, or KI). Notably, position 8 along this peptide has been shown to be an important position for T cell recognition and T cell activation (Maryanski et al., 1993; Kessler et al., 1998), and thus an important residue to study the impact of potential variant amino acid changes. Herein, we show that single amino acid changes to the T cell receptor (TCR) contact residue, position 8 (lysine), of the SYIPSAEKI epitope can lead to a loss of recognition by T cells. We further assess the impact of utilizing diverse adjuvants, representing both inflammatory and non-inflammatory adjuvant systems, to affect the magnitude and breadth of cross-reactivity of the immune responses.

## Materials and Methods

### Animals

BALB/c mice (6–8 weeks old) were purchased from Monash Animal Services (MAS, Melbourne, VIC, Australia). All animals were used under ethics approval by the Alfred Medical Research and Education Precinct (AMREP) Animal Ethics Committee.

### *Ex Vivo* DC Pulsing with Peptide

Hematopoietic stem cells were harvested from the femur and tibia of BALB/c mice, and seeded at 0.5 × 10^6^ cells/ml in complete media (CM) containing RPMI 1640 supplemented with 10% fetal calf serum (FCS, Gibco, Life Technologies), 100 units/ml penicillin, 100 μg/ml streptomycin (Gibco), 2 mM L-Glutamine (Gibco), 1 M Hepes, and 0.1 mM 2-mercaptoethanol (Sigma–Aldrich, St Louis, MO, USA), and additionally a final of 10 ng/ml Granulocyte-Macrophage Colony-Stimulating Factor (GM-CSF) (PeproTech, Rocky Hill, NJ, USA) and 5 ng/ml Interleukin 4 (IL-4) for 6 days (note that half of the media was replaced with replenished cytokines on day 3) to induce the differentiation of DCs. On day 6, DCs were pulsed with 5 μg/ml SYIPSAEKI peptide for 1–2 h in the same culture media, and then washed with phosphate buffered saline (PBS) and re-suspended at 1 × 10^7^ cells/ml in PBS for immunizations.

### Peptide Conjugation to Nanoparticles

Nanovaccine formulations were prepared by conjugation of peptides SYIPSAEKI, SYIPSAERI, SYIPSAEVI, SYIPSAEAI, SYIPSAEEI, or SYIPSAEDI to carboxylated PSNPs (Polysciences Inc., Warrington, PA, USA) based on the previously outlined method (Xiang et al., 2013; Wilson et al., 2015). Briefly, PSNPs (~40–50 nm) at a final of 1% solids were activated using a 2-*N*-morpholino-ethanesulfonic acid (MES; 50 mM final, pH = 7) buffered solution of 1-ethyl-3-(3-dimethylaminopropryl) carbodiimide hydrochloride (EDC; 4 mg/ml final, Sigma–Aldrich), together with the malaria peptides (SYIPSAEKI or the variants SYIPSAERI, SYIPSAEVI, SYIPSAEAI, SYIPSAEEI, and SYIPSAEDI, Mimotopes, Melbourne, VIC, Australia; 1 mg/ml final) on a rotary wheel, for 4 h at room temperature (RT). Following incubation, the conjugation reaction was stopped and unbound reactive sites coated by the addition of glycine (7 mg/ml final, Sigma–Aldrich) and further incubated as above for 30 min. Unconjugated peptide and excess glycine and other reagents were removed by dialysing in dialysis membrane (10–14 kDa molecular weight cutoff; Viskase, Darien, USA), against PBS (pH = 7.2–7.4) overnight at 4°C. Conjugation efficiency was determined by Bicinchoninic Assay (BCA^TM^; Thermo Fisher Scientific, Rockford, IL, USA) following manufacturer’s instruction. Final size of the formulation was determined by dynamic light scattering instruments (Zetasizer; Malvern Instruments, Worcestershire, UK).

### Vaccine Adjuvants and Immunizations

Vaccines consisted of the following formulations in PBS; SYIPSAEKI (final 5 μg/ml) pulsed DCs (1 × 10^6^ cells/mouse in 100 μl PBS) with or without the addition of PSNPs (50 μl at 2% solids mixed in); SYIPSAEKI (final 25 μg/mouse) mixed with the adjuvants Montanide ISA 720 (70% v/v with PBS; Tall Bennet Group, USA), or Polyinosinic-polycytidylic acid sodium salt (Poly I:C; 25 μg/mouse final, Sigma–Aldrich). Nanovaccines contained either SYIPSAEKI (~25 μg/mouse) simply mixed with PSNPs (~1% final solids), or peptides covalently conjugated to the PSNPs, including SYIPSAEKI or the variant peptides SYIPSAERI, SYIPSAEVI, SYIPSAEAI, SYIPSAEEI, and SYIPSAEDI (target 25 μg/mouse, ~0.65–1.47% final solids to achieve target peptide loading per mouse). Mice were immunized with respective formulations, either once, or twice, 2 weeks apart, intradermally at the base of tail. Approximately 13–14 days after the last immunization (unless otherwise stated) mice were humanely euthanized and splenocytes harvested and assessed for IFN-γ production by ELISpot assay.

### ELISpot

Antigen specific and cross reactive CD8 T cell responses were assessed by IFN-γ ELISpot assay. Ninty-six well multiscreen plates (MSIP; Millipore, Billerica, USA) were coated with 5 μg/ml anti-mouse IFN-γ (AN18; MABTech, Stockholm, Sweden), 100 μl/well in PBS, and incubated at 4°C overnight. Following incubation, all wells were washed five times with PBS and blocked with CM for a minimum of 1 h at 37°C. Splenocytes harvested from immunized mice were added to wells in triplicate (1 × 10^7^ cells/ml, 50 μl/well), and co-incubated with the different recall antigens (SYIPSAEKI, SYIPSAERI, SYIPSAEVI, SYIPSAEAI, SYIPSAEEI, SYIPSAEDI; Mimetopes, Melbourne, Australia; final 2.5 μg/ml), as well as a media alone control, and concanavalin A (ConA; Amersham Biosciences, Uppsala, Sweden; final 1 μg/ml) as positive control. Splenocytes and antigens were co-incubated in CM at 37°C, with 6% C0_2_ for 12–16 h. Following incubation, plates were washed with PBS and the anti-IFN-γ biotinylated detection antibody was added (R4-6A2-biotin, MABTech; 1 μg/ml final in PBS/0.5% FCS), 100 μl/well, and incubated at RT for 2 h. Plates were washed as above before addition of streptavidin-alkaline phosphate enzyme conjugate (ALP; final 1 μg/ml in PBS/0.5% FCS), 100 μl/well, for 1.5 h at RT. Plates were washed with PBS as above and given a final wash with reverse osmosis (RO) water. Spots were developed using an AP colorimetric kit (Bio-Rad, Philadelphia, PA, USA), following manufacturer’s instructions. Plates were left to dry overnight at RT before spots were counted using an AID ELISpot reader system (AutoImmune Diagnostika GmbH, Germany).

### Binding Assay

For determination of peptide binding ability to MHC class I (MHCI) (H2-Kd), a binding assay using a MHCI (H2-Kd) transfected RMA-S cell line was performed. Briefly, RMA-S-Kd cells were cultured in CM further supplemented with 0.8 mg/ml G418 disulphide salt (Sigma–Aldrich), until confluent. Cells were seeded into 48 well plates (0.5 × 10^6^ cells/ml) and incubated at 28–29°C overnight with 6% C0_2_, allowing cells to express H2-Kd. Following incubation peptides (SYIPSAEKI, SYIPSAERI, SYIPSAEVI, SYIPSAEAI, SYIPSAEEI, and SYIPSAEDI) were added in triplicate wells (at final concentrations of 0, 0.025, 0.25, and 2.5 μg/ml), and further incubated at 28–29°C with 6% C0_2_ for 1 h. Plates were then transferred to 37°C, 6% C0_2_, for an additional 3 h so that empty H2-Kd molecules could be recycled.

Cells were transferred to 96 well ‘V’ bottom plates for flow cytometry staining and stained with biotinylated anti-H2-Kd mAb (clone SF1-1.1; Biolegend, San Diego, CA, USA), or isotype control biotinylated anti-mouse IgG2aκ (Biolegend), at pre-determined dilutions, in PBS/2% FCS (30 μl/well) for 15 min at RT. Following incubation cells were washed with PBS/2% FCS (100 μl/well), and centrifuged before addition of streptavidin-AF700 (Thermo-Fisher) in PBS/2% FCS (30 μl/well) for 15 min at RT. Stained cells were given a final wash with PBS/2% FCS and fixed with 1% (v/v) paraformaldehyde (PFA; Sigma–Aldrich). Cells were acquired using an LSRII flow cytometer (BD Biosciences, USA), located at the AMREP Flow Cytometry Core Facility (Melbourne, Australia). Data was analyzed using FlowJo software (version 10; Treestar, USA).

### Statistics

Statistical analysis was conducted using two way analysis of variance (ANOVA), with *post hoc* Dunnetts (or Tukey where applicable), multiple comparison tests, using Graphpad Prism software (v.6. San Diego, CA, USA). Statistical significance was set as *p* < 0.05. Values are expressed as mean ± standard deviation (SD), and group sizes are indicated in the figure legends.

## Results

### *Ex Vivo* Peptide Pulsed DCs as Natural Adjuvants Induce Low Levels of Cross Reactivity to APLs of the CD8 Epitope SYIPSAEKI

APLs of the *P. berghei* immunodominant CD8 T cell epitope SYIPSAEKI (herein termed KI) were created by substituting the lysine at position 8 with amino acids carrying different charges. Five altered peptides were created; SYIPSAERI (RI), arginine substitution at position 8; SYIPSAEVI (VI), valine substitution; SYIPSAEAI (AI), alanine substitution; SYIPSAEEI (EI), glutamic acid substitution; and SYIPSAEDI (DI), aspartic acid substitution. Additionally, the substituted amino acids alter the charge of the position 8 amino acid, KI and RI carry a positive side chain charge, VI and AI carry a neutral side chain charge, and EI and DI carry a negative side chain charge. Notably, side chain charge may not alter substantially the overriding charge of the peptide. Cross reactivity to these APLs was initially examined using various adjuvant systems immunized with the index peptide KI.

*Ex vivo* peptide pulsed DCs are naturally immunogenic and are advantageous due to their ability to effectively prime antigen specific T cells, without requiring an additional adjuvant. Bone marrow was extracted from BALB/c mice and cultured for 6 days, with the cytokines GM-CSF and IL-4, and cultured DCs were pulsed with KI for 1–2 h prior to immunization (DCs/KI). Previous studies have shown that non-inflammatory nanoparticles (PSNPs) further enhance immune responses when mixed with micro-particulate vaccines (Fifis et al., 2004; Xiang et al., 2015). Therefore, KI pulsed DCs were additionally co-injected with PSNPs alone (DCs/KI + PSNPs), to examine if a potential similar enhancement would also have an effect on the pattern of cross reactivity to APL variants. BALB/c mice were immunized, once with the above formulations, and 35 days after the immunization splenocytes were harvested and analyzed for IFN-γ production via ELISpot assay.

**FIGURE 1:**
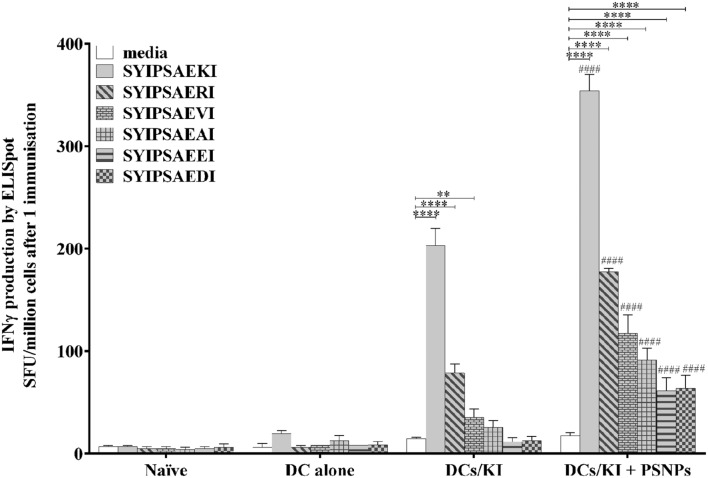
**Cross reactivity to APLs when adjuvanted by KI pulsed DCs, with or without nanoparticles (PSNPs) co-injection.** BALB/c mice were immunized once with naïve control (PBS alone), DCs alone (1 × 10^6^ cells in PBS), or peptide pulsed DCs (1 × 10^6^ cells pulsed with 5 μg/ml KI, DCs/KI), with or without co-injected PSNPs (1 × 10^6^ cells pulsed with 5 μg/ml KI co-injected with a final 0.67% PSNPs, DCs/KI + PSNPs). Thirty five days after the immunization splenocytes were harvested and antigen specific or cross reactive IFN-γ production was measured via ELISpot assay. Data shown as mean ± SD of SFU/million cells (pooled cells from four mice per group) from triplicate wells. **p < 0.01, ****p < 0.0001, ####*p* < 0.0001 between DCs/KI and DCs/KI + PSNPs groups.

**Figure 1** shows that KI pulsed DCs induced significantly higher levels of KI specific IFN-γ to the peptide itself, compared to the media alone control (*p* < 0.0001, **Figure 1**), and a moderate level of cross reactivity to the other APL variants, in particular significant responses above media for RI (*p* < 0.0001), and VI (*p* < 0.01). The ranking of APL responses showed that RI (positive charge at position 8, the same charge as the lysine in KI) was the most cross reactive, followed by VI and AI (neutral charge at position 8), with EI and DI (negative charge at position 8) the least cross reactive to the index peptide KI (**Figure 1**). Interestingly, KI pulsed DCs co-injected with PSNPs showed significantly higher KI specific IFN-γ responses for all peptides, apart from media alone, compared to the DCs/KI group (*p* < 0.0001 for each peptide), as well as significantly increased magnitude of responses, compared to media, to all five cross reactive APLs tested (*p* < 0.0001 for all APLs). Notably, the pattern of IFN-γ response to the APLs remained the same, suggesting that PSNPs may not be directly affecting the breadth of the T cell response, but may enhance the number of reactive T cells, which could proportionally enhance the magnitude of responses.

### Limited APL Cross Reactivity is not a Consequence of Lack of Peptide Binding to MHCI

**FIGURE 2:**
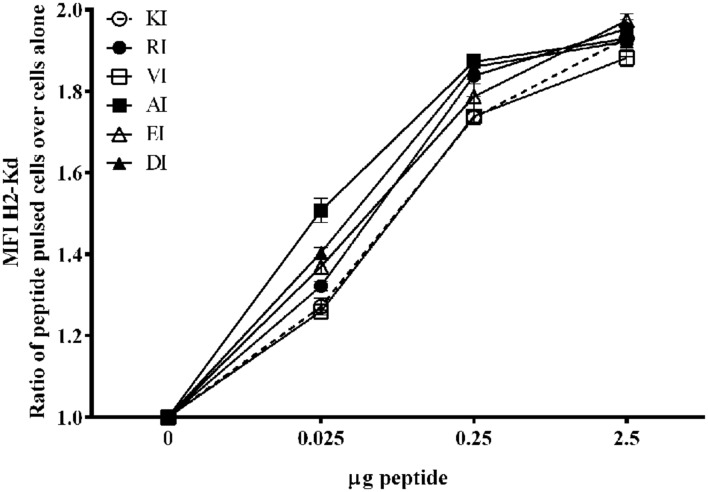
**Binding ability of the index peptide, KI, or APLs to the MHC molecule H2-Kd.** H2-Kd transfected RMA-S cells were cultured *in vitro* overnight to allow expression of the MHC, and subsequently co-cultured KI, or with each of the altered peptides, in triplicate at three different concentrations, for peptide-MHC complex stabilization at the cell surface. Cells were stained with anti-mouse H2-Kd and the mean fluorescent intensity (MFI) of H2-Kd for each condition was determined (0 is equivalent to the MFI for RMA-S-H2-Kd cells alone). Data shown as MFI of H2-Kd expression (Ratio of peptide pulsed cells over cells alone) ±SD from triplicate wells. Dotted line represents the index peptide KI.

The low level of cross reactive responses to certain epitopes, particularly DI and EI, could be explained if these APL variants had a lower binding capacity to MHCI than KI. To assess this formally, we performed a binding assay which utilizes the cell line, RMA-S, deficient in MHCI loading molecules, which has been transfected with H2-Kd, creating a cell line reported to have a heat sensitive expression of MHCI. In temperature ranges of 26–30°C H2-Kd is expressed on the cell surface, allowing peptide binding and stabilization of the peptide-MHC complex (Ljunggren et al., 1990; Rock et al., 1992). At higher temperatures (37°C) empty MHC molecules start to be recycled back into the cell, unless they are stabilized on the surface by exogenously binding peptide. Detection of surface peptide-MHCI complexes is achieved through flow cytometry staining for H2-Kd. Of interest to this study was the relative comparison of peptide binding ability of the APLs to the index peptide, KI. As can be seen in **Figure 2**, all variant peptides achieved an equal, or greater, level of binding compared to KI at all three concentrations of peptide loading tested (0.025, 0.25, and 2.5 μg/ml). This suggests that the limited cross reactive responses observed were not due to a lack of binding ability to MHCI for these variant peptides.

### The Adjuvant Montanide Elicits a Similar Pattern of Cross Reactivity Compared to Peptide Pulsed DCs

A high magnitude of antigen specific IFN-γ responses have previously been shown to the KI peptide *in vivo* by using the conventional inflammatory adjuvant Montanide, with lower responses inducible with Poly I:C (Wilson et al., 2015). To examine the cross reactivity of the five APLs mentioned above we examined the responses induced using Montanide or Poly I:C as the vaccine adjuvant. BALB/c mice were immunized either once, or twice, with the peptide alone (KI alone), or mixed with Montanide or Poly I:C (Montanide + KI and Poly I:C + KI, respectively), and 14 days after the last immunization IFN-γ production was assessed via ELISpot assay.

**FIGURE 3:**
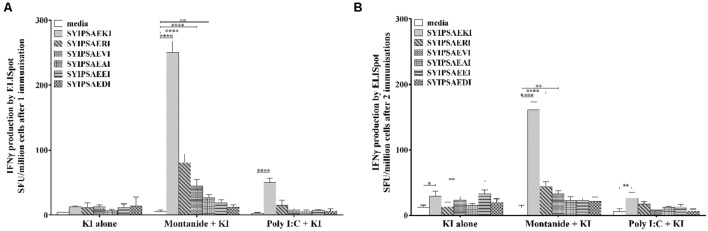
**Cross reactivity to APLs of the peptide SYIPSAEKI induced by the conventional adjuvants Montanide or Poly I:C.** BALB/c mice were immunized once **(A)**, or twice **(B)** with KI peptide alone (25 μg /mouse), or mixed with Montanide (25 μg peptide mixed with 70% v/v Montanide in PBS /mouse, Montanide + KI) or Poly I:C (25 μg peptide and 25 μg Poly I:C /mouse, Poly I:C + KI). Fourteen days after the last immunization splenocytes were harvested and antigen specific or cross reactive IFN-γ production was measured via ELISpot assay. Data shown as mean ± SD of SFU/million cells (pooled cells from 3 to 4 mice per group) from triplicate wells. *p < 0.05, **p < 0.01, ****p < 0.0001

After one immunization (**Figure 3A**), Montanide + KI induced the highest IFN-γ production to the index epitope, KI, significantly higher compared to the media alone background (*p* < 0.0001). Furthermore, moderate compared to KI, but significant, cross reactivity was seen with this group to the APLs RI and VI (*p* < 0.0001), as well as to AI (*p* < 0.01), similar to the pattern observed with *ex vivo* pulsed DCs. Poly I:C + KI only induced significant levels of IFN-γ response to KI (*p* < 0.0001), showing a corresponding decreased ability to induce detectable cross reactive T cells. A further boost immunization displayed a similar pattern of results for the Montanide + KI group, with a significantly high amount of KI specific CD8 T cell responses (*p* < 0.0001), and low to moderate cross reactivity to the APLs, in particular significant cross reactivity to RI (*p* < 0.0001), and VI (*p* < 0.01), with overall magnitude of responses lower, but ranked similar, compared to one immunization (**Figure 3B**). The boost immunization did not enhance responses induced by the Poly I:C + KI group.

### Conjugated PSNPs Induce Little Cross Reactivity to APLs of the CD8 Epitope SYIPSAEKI

**FIGURE 4:**
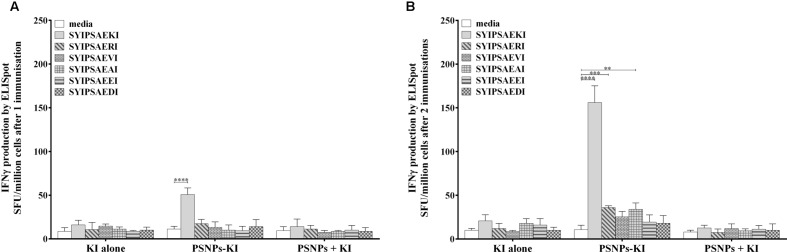
**Cross reactivity to APLs of the peptide SYIPSAEKI induced by nanovaccine systems.** BALB/c mice were immunized once **(A)**, or twice **(B)** with KI peptide alone, or either mixed with, or conjugated to, PSNPs (25 μg peptide, 1% PSNPs /mouse for both mixed in and conjugated groups). Fourteen days after the last immunization splenocytes were harvested and antigen specific or cross reactive IFN-γ production was measured via ELISpot assay. Data shown as mean ± SD of SFU/million cells (pooled cells from three mice per group) from triplicate wells. **p < 0.01, ***p < 0.001, ****p < 0.0001

Previously it has also been observed that peptide conjugated to, but not mixed with, PSNPs induces high levels of KI specific CD8 T cell responses (Wilson et al., 2015). Following this, cross reactivity to APLs of KI were investigated after one or two immunizations with KI mixed with, or conjugated to, PSNPs. After one immunization, there were low numbers of KI specific CD8 T cell responses induced by the PSNPs-KI conjugated group, albeit significant compared to media alone (*p* < 0.0001), and cross reactive CD8 T cell activity was not detectable (**Figure 4A**).

A boost immunization enhanced the KI specific IFN-γ response to the PSNPs-KI group approximately threefold larger compared to one immunization, with a slight boost to the small cross reactive responses, resulting in the detection of significant responses also to RI (*p* < 0.001), and AI (*p* < 0.01, **Figure 4B**), compared to media alone. Overall little cross reactivity was seen to the APL variants (**Figure 4B**), albeit the ranking of cross reactivity to the different variants remained consistent, regarding side chain charge, to that seen with Montanide and *ex vivo* pulsed DCs. Also consistent with previous findings, the peptide needed to be conjugated to the PSNPs to elicit a response, as the peptide alone or peptide mixed with PSNPs did not induce detectable KI specific or cross reactive responses after a single or boost immunization (**Figures 4A,B**).

### The APL DI Conjugated to PSNPs Induces a Broader Pattern of Cross Reactive Responses Compared to that Induced by the Other Variants

Across the previous adjuvants examined the most cross reactivity was observed to APLs carrying a positive or neutral side chain charge. The lowest levels of cross reactive responses were consistently seen to the APLs carrying a negative charged residue at position 8, namely EI and DI. To test whether one of the APL variants could possibly enhance the peptide specific or cross reactive responses, we further conjugated each APL to PSNPs using the method described previously, and examined the pattern of response by IFN-γ ELISpot 13 days after one immunization. Nanovaccine formulations were chosen as the preferred carrier system, to investigate whether an improvement in cross reactivity could be seen when immunizing with a variant compared to the index KI. Furthermore, there are advantages to using non-inflammatory adjuvants/carriers in vaccine formulations.

**FIGURE 5:**
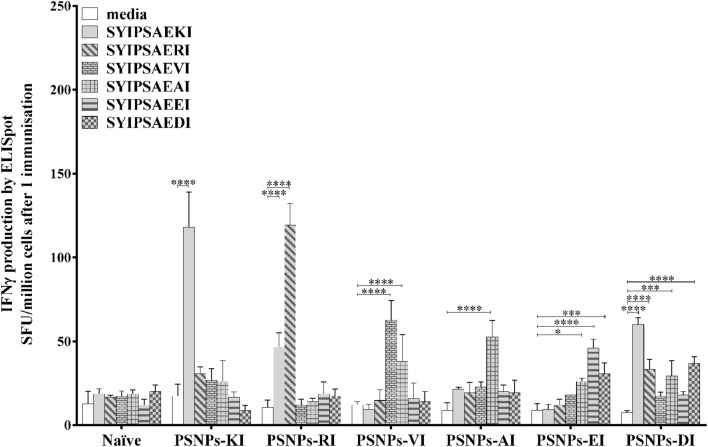
**Antigen specific and cross reactive CD8 T cell responses induced by APL conjugated PSNPs.** BALB/c mice were immunized once with the conjugated APLs SYIPSAEKI (25 μg peptide, 0.76% PSNPs/mouse), SYIPSAERI (25 μg peptide, 0.65% PSNPs/mouse), SYIPSAEVI (25 μg peptide, 0.86% PSNPs/mouse), SYIPSAEAI (25 μg peptide, 1.09% PSNPs/mouse), SYIPSAEEI (25 μg peptide, 1.47% PSNPs/mouse), and SYIPSAEDI (25 μg peptide, 1.02% PSNPs/mouse) conjugated to PSNPs. Thirteen days after the immunization splenocytes were harvested and antigen specific or cross reactive IFN-γ production was measured via ELISpot assay. Data shown as mean ± SD of SFU/million cells (pooled cells from 3 mice per group) from triplicate wells. *p < 0.05, ***p < 0.001, ****p < 0.0001.

Consistent with previous data, after one immunization the PSNPs-KI immunized group showed significant IFN-γ production to the KI epitope (*p* < 0.0001, **Figure 5**), however, no significant cross reactive responses were observed to other variants. Immunization with PSNPs-RI induced significant self-reactive responses to RI, as well as significant cross reactivity to KI (*p* < 0.0001). PSNPs-VI induced reactivity to VI, and cross reactivity to AI (*p* < 0.0001), but no cross reactivity to KI. Likewise immunizing with PSNPs-AI showed no reactivity to the index KI, or any other epitope, besides significant reactivity to itself (*p* < 0.0001). Interestingly, the PSNPs-EI group induced significant CD8 T cell responses to EI itself (*p* < 0.0001), as well as DI (*p* < 0.001), and AI (*p* < 0.05), but no significant responses were detected to VI, RI, or KI. Surprisingly, PSNPs-DI immunization induced significant reactivity to DI (*p* < 0.001), and cross -reactivity to AI (*p* < 0.001), RI (*p* < 0.0001), and KI (*p* < 0.0001), with the response to KI even greater than that to DI itself. Of the variants tested, only two, RI and DI, were identified as being cross-reactive to the index peptide epitope KI in addition to the response to the priming epitope itself. This implies a ‘one-way’ cross reactivity, where PSNPs-KI cannot induce a significant cross reactive response to DI or RI after one immunization, though conversely, PSNPs-DI and PSNPs-RI can induce a cross reactive response to KI. Overall, PSNPs-DI induced a substantially different pattern of cross-reactivity, to RI, AI, and KI, after one immunization compared to the responses induced by the other conjugated APLs. It would be of interest to investigate in future the breadth of response to DI incorporated with other powerful adjuvant systems, considering that in this study the adjuvant system employed did not substantially alter reactivity, though impacted the overall magnitude of response.

## Discussion

The current study presents an *in vivo* model to investigate how polymorphism and variability in protective CD8 T cell epitopes from infectious pathogens, such as malaria, can possibly limit the ability of the vaccines to eradicate them. We have focused on modifications of amino acid position 8 of the protective CD8 T cell epitope of *P. berghei*, KI, since this position has been previously demonstrated to physically interact with affinity modulating regions of a cognate T cell receptor (TCR; Maryanski et al., 1993; Kessler et al., 1998; Guillaume et al., 2003). Furthermore, modifications at this amino-acid position did not alter binding affinity to the MHCI molecule (**Figure 2**). This model was therefore used to investigate for the first time whether the use of different adjuvants, including conventional inflammation promoting adjuvants and novel non-inflammatory nanoparticle based carrier systems, could broaden the repertoire of T cells induced by vaccines. Additionally, we explored whether these adjuvants could potentially be used to generate broad patterns of cross-reactivity capable of recognizing populations of highly variable pathogens, simulating those naturally present in malaria endemic regions.

Vaccination with the immunodominant CD8 T cell epitope KI indicated that reactivity to APLs was unable to be enhanced by the various adjuvant systems. The results showed that the induced responses were largely specific to the immunizing KI epitope itself, with only limited cross-reactivity observed to other variants. The low to moderate level of reactivity that was observed to variant epitopes of KI was not due to a simple lack of binding ability, as all tested variants bound as strongly, or even stronger, to MHCI than the index peptide KI. The cross reactivity of KI was focused primarily on the variant RI, which was the most highly related variant to KI (both positively charged at position 8). Furthermore, the KI induced responses failed to elicit substantial cross-reactivity to other variants with a neutral (AI and VI) or negative (EI and DI) charge at position 8, regardless of the adjuvant systems used. Cross-reactive responses, when found, were directly related to the magnitude of the immune response induced to the index KI T cell epitope. Importantly, there was no direct relationship between the intrinsic nature of the adjuvant system being utilized, whether this was based on vaccines with water-in-oil emulsions (Montanide), ‘danger signals’ (Poly I:C), non-inflammatory nano-carriers (PSNPs) or *ex vivo* peptide pulsed DCs. These data sets indicate that for vaccines to be truly broadly cross reactive, it is necessary to look beyond enhancing the magnitude of the index response. Simply modifying the type of adjuvant is unlikely to modify the process of priming or boosting immunity sufficiently to provide broad cross reactive immune responses.

The fact that DI and EI were unable to elicit strong effector T cell reactivity *in vitro*, from cells primed with KI, could indicate that the naïve T cell repertoire of the animals was inherently limited in recognizing such variants, for example, due to holes in the TCR repertoire produced from cross reactivity to self-antigens. Alternatively, it could have meant that these variants may be immunogenic themselves, but did not cross react with KI because their recognition requires a different set of TCRs from that induced by these variants. To distinguish between these possibilities, we immunized with each APL conjugated to the nanoparticle vaccine carrier PSNPs. What we found was that EI and DI were indeed immunogenic, eliciting antigen specific T cells, and hence the inability of KI to induce cross reactive responses was not due to holes in the TCR repertoire. Moreover, DI was capable of inducing a broader pattern of T cell reactivity, inducing KI specific CD8 T cells to comparable, or even higher levels as to its own DI specific T cells. This “one-way” cross reactivity observation was specific to the DI and RI variants, whilst the other immunized APLs predominantly induced homologous responses to the immunization peptide itself. Though RI immunized mice elicited CD8 T cell reactivity to RI and KI, DI immunized mice were the only group capable of inducing cross-reactive responses that were broader than the antigen specific and index peptide reactive responses induced to the immunized epitope. There are intriguing consequences to the identification of the existence of one-directional cross reactivity. For polymorphic pathogens, it suggests that some natural variants may have the advantage of not being recognized by immune responses induced by other variants, whilst being able themselves to elicit immunity which eliminates other strains in the same populations.

For vaccine development, these findings show it may be possible in future to rationally re-engineer polymorphic antigens to enable them to recognize multiple relevant variants in a given population of pathogens. Thus acknowledging the fact that there is extensive research required before the use of re-engineered polymorphic antigens can commonly be used in vaccine design, especially for malaria vaccine development, which already carries a host of inherent challenges. Moreover, although murine models cannot directly be applied to human studies, the basic knowledge and understanding of interactions between APLs and reactivity induced by modifying the peptide, or by using various adjuvant systems, could be tested against known human variants. Importantly, this study has shown that for vaccine design it is relevant to consider immune responses elicited to APLs, and that it is possible to alter the response by modifying polymorphic epitopes, instead of simply varying the adjuvant system used. Given that broader responses were only seen with certain variants, it would be crucial to screen potential targets for their reactivity to evaluate their value as a vaccine candidate. We envisage such approaches would be able to be taken to tackle the many polymorphic pathogens for which there are still no effective broadly reactive vaccines, and particularly help the development of potent liver-stage malaria vaccines.

## Author Contributions

KW designed and performed experiments, analyzed data, wrote the manuscript. SX helped design experiments and reviewed the manuscript. MP conceived the project, planned experiments, analyzed data, funded the project and wrote the manuscript.

## Conflict of Interest Statement

The authors declare that the research was conducted in the absence of any commercial or financial relationships that could be construed as a potential conflict of interest.
